# Association Between Sex and Immune Checkpoint Inhibitor Outcomes for Patients With Melanoma

**DOI:** 10.1001/jamanetworkopen.2021.36823

**Published:** 2021-12-02

**Authors:** Se Ryeong Jang, Nikita Nikita, Joshua Banks, Scott W. Keith, Jennifer M. Johnson, Melissa Wilson, Grace Lu-Yao

**Affiliations:** 1 Franchise Health Economics and Market Access, Ethicon, Raritan, New Jersey; 2 College of Population Health, Thomas Jefferson University, Philadelphia, Pennsylvania; 3 Department of Medical Oncology, Sidney Kimmel Medical College, Thomas Jefferson University, Philadelphia, Pennsylvania; 4 Sidney Kimmel Cancer Center, Thomas Jefferson University, Philadelphia, Pennsylvania; 5 Division of Biostatistics, Department of Pharmacology and Experimental Therapeutics, Sidney Kimmel Medical College, Thomas Jefferson University, Philadelphia, Pennsylvania; 6 St Luke’s Cancer Center, Department of Oncology, St Luke’s University Health Network, Easton, Pennsylvania

## Abstract

**Importance:**

Immune checkpoint inhibitors (ICIs) have revolutionized melanoma treatment and are now standard of care. Although sex is associated with immune function and immune-related diseases, the interaction between sex and ICIs is understudied.

**Objective:**

To examine whether cancer immunotherapy effectiveness varies between female and male patients with advanced melanoma treated with either nivolumab plus ipilimumab combination therapy or anti–programmed cell death protein 1 (PD-1) therapy (namely, pembrolizumab or nivolumab).

**Design, Setting, and Participants:**

The study population consisted of 1369 older adults (aged ≥65 years) with a record of melanoma diagnosis from January 1, 1991, to December 31, 2015, in the Surveillance, Epidemiology, and End Results–Medicare linked database. Patients with a diagnosis of stage III or stage IV melanoma and a claims record showing nivolumab plus ipilimumab combination therapy or anti–PD-1 therapy (ie, pembrolizumab or nivolumab) as their last type of ICI prescribed were included in the analyses. Patients were followed up through December 31, 2017, for the overall survival analysis. Statistical analysis was performed from September 19, 2019, to February 20, 2021.

**Exposures:**

Sex, last prescribed ICI, and prior use of ipilimumab.

**Main Outcomes and Measures:**

The primary outcome was overall survival, defined as time from the index date until death from any cause, with patients censored at the end of the study (December 31, 2017). Cox proportional hazards regression modeling was used to examine the association of sex with ICI outcomes while adjusting for prior use of ipilimumab, age at ICI initiation, Charlson Comorbidity Index, cancer stage at the time of diagnosis, and autoimmune disease diagnosis.

**Results:**

Among the 1369 patients in the study (982 men [71.7%]; median age, 75 years [IQR, 69-82 years]), the outcome of nivolumab plus ipilimumab combination therapy depended on sex (Wald χ^2^ = 9.48; *P* = .009 for interaction). The mortality hazard ratio (HR) for women with prior ipilimumab use receiving combination therapy was 2.06 times (95% CI, 1.28-3.32; *P* = .003) higher than their male counterparts. No significant difference was observed between women and men receiving anti–PD-1 therapy with (HR, 0.97 [95% CI, 0.68-1.38]; *P* = .85) or without prior ipilimumab use (HR, 0.85 [95% CI, 0.67-1.07]; *P* = .16). For women with prior ipilimumab use, combination therapy was associated with 2.82 times higher mortality hazards than anti–PD-1 therapy (95% CI, 1.73-4.60). No statistically significant difference was seen in mortality risk between anti–PD-1 therapy and combination therapy for men.

**Conclusions and Relevance:**

This cohort study suggests that female patients with advanced melanoma may not benefit as much from combination ICIs as male patients would. Tumor mutation burden or estrogen level may serve as an important biomarker associated with ICI response in metastatic melanoma.

## Key Points

**Question:**

Does the effectiveness of cancer immunotherapy vary between female and male patients with advanced melanoma?

**Findings:**

In this population-based cohort study that included 1369 patients 65 years of age or older with advanced melanoma, a significant sex difference in overall mortality was seen among patients treated with nivolumab plus ipilimumab combination immunotherapy, with women having a 2-fold higher mortality risk than their male counterparts.

**Meaning:**

This study suggests that the sex of the patient must be considered when designing a treatment strategy for patients with metastatic melanoma to optimize outcomes.

## Teaser

This cohort study examines whether cancer immunotherapy effectiveness varies between female and male patients with advanced melanoma treated with either nivolumab plus ipilimumab combination therapy or anti–programmed cell death protein 1 therapy.

## Introduction

Immune checkpoint inhibitors (ICIs) are now the mainstay of treatment for advanced melanoma. To date, the US Food and Drug Administration has approved the following 3 ICIs for the treatment for advanced melanoma: ipilimumab (cytotoxic T-lymphocyte-associated antigen 4 [CTLA-4] inhibitor), pembrolizumab, and nivolumab (programmed cell death protein 1 [PD-1] inhibitors). These ICIs have improved the prognosis of metastatic melanoma by reversing effector T-cell dysfunction and exhaustion, thereby reinvigorating antitumor immune response.^1,2,3^ Acting at different points in the T-cell response pathway, the CTLA-4 inhibitor and the PD-1 inhibitor could generate a synergistic effect when used in combination.^4,5^ For instance, nivolumab plus ipilimumab resulted in the highest 5-year survival rate (52%) compared with nivolumab alone (44%) and ipilimumab alone (26%) in the recent update of the phase 2 CheckMate 067 trial.^6^ However, the combination therapy is associated with high toxicity, and the overall ICI response rate remains low, at 30% to 40%.^1,5,7,8^ It is therefore imperative to identify factors associated with drug responses to optimize ICI strategies.

Biological markers such as sex may serve as an important factor associated with ICI response in patients with melanoma based on the following observations. First, the incidence of melanoma varies between women and men by age. Although melanoma is predominantly found in men older than 50 years of age, female patients make up 60% of patients younger than 50 years with melanoma.^9^ The comparatively low incidence of melanoma among postmenopausal women coupled with the high incidence of melanoma among women in their reproductive years suggests that sex hormones, such as estrogen, may play a role in the development of melanoma.^10^ Second, faster clearance of pathogens and greater vaccine effectiveness are seen in women.^11,12^ Third, women are 4-fold more susceptible to autoimmune diseases than men.^11,12^ These observations illustrate that women mount stronger immunologic responses to foreign and self-antigens and may respond to ICIs differently than their male counterparts.^13^

It is uncertain whether the effectiveness of ICI-based immunotherapy treatment of metastatic melanoma varies by sex. Therefore, we conducted a population-based cohort study to address this question.

## Methods

### Study Design and Data Source

This observational cohort study consists of Medicare patients receiving either a PD-1 inhibitor (nivolumab or pembrolizumab) or nivolumab plus ipilimumab combination therapy for advanced melanoma. The main data source was Surveillance, Epidemiology, and End Results (SEER)–Medicare linked data, including patients who received a diagnosis of melanoma between 1991 and 2015.

The SEER-Medicare linked files include clinical data from population-based cancer registries and health encounter records for those with fee-for-service insurance. As of 2017, the SEER program covered approximately 35% of the US population, with a 98% case ascertainment rate.^14,15^ Our patient cohort represents approximately 60% of Medicare beneficiaries.

The research question, study design, and end points were all prespecified in the research protocol, as recommended in the Strengthening the Reporting of Observational Studies in Epidemiology (STROBE) reporting guidelines.^16^ The study was approved by the Thomas Jefferson University institutional review board, which waived the need for obtaining informed patient consent because the data were deidentified, and conformed to the data user agreement.

### Study Population

Patients with a diagnosis of melanoma (*International Classification of Diseases, Ninth Revision* codes 172.0-172.9; *International Statistical Classification of Diseases and Related Health Problems, Tenth Revision* codes C43.0-C43.9) in the SEER registry between January 1, 1991, and December 31, 2015, were identified. We excluded those who received a diagnosis after death, were enrolled in health maintenance organizations, or had no Medicare Part A or B coverage during the study period. We further restricted the patient cohort by including only those who had a claims record of nivolumab (Healthcare Common Procedure Coding System [HCPCS] code J9299), pembrolizumab (HCPCS code J9228), or nivolumab plus ipilimumab combination therapy (presence of both HCPCS codes on the same date). Claims records for pembrolizumab and nivolumab were available from January 1 to December 29, 2016, while records for ipilimumab prescriptions were available from January 1, 2012, to December 29, 2016. Given that claims for nivolumab were made starting in 2016 in our data set, prescriptions for nivolumab plus ipilimumab combination therapy were only available for 2016. The difference in the availability of claims records for each treatment reflects the US Food and Drug Administration approval dates for each (ipilimumab, 2011; pembrolizumab and nivolumab, 2014; nivolumab plus ipilimumab combination, 2015).

### Exposure and Outcome Definitions

The main exposure of interest was the last ICI administered. We classified treatment groups by the last prescription for ICI(s) as captured in Medicare’s claims files (ie, the Outpatient file and the National Claims History file). We followed up with patients from the first prescription date for the last ICI captured in their claims file until death or last date of vital status available.

**Figure 1.**
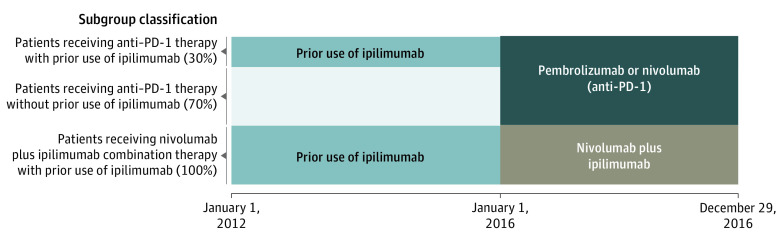
Breakdown of Patient Cohort PD-1 indicates programmed cell death protein 1.

Our data indicated that 30.0% (362 of 1204) of patients receiving anti–PD-1 therapy had prior ipilimumab use, whereas 100% of patients receiving combination therapy had prior ipilimumab use. To control for the significant imbalance in history of ipilimumab use, we further classified the patient cohort into the following subgroups: anti–PD-1 therapy without prior ipilimumab use, anti–PD-1 therapy with prior ipilimumab use, and combination therapy with prior ipilimumab use (Figure 1).

We used the Patient Entitlement and Diagnosis Summary File from the SEER Program and Medicare claims to identify the following variables: sex, age at diagnosis and at index time (initiation date of the last type of ICI administered), year of diagnosis, SEER region, cancer stage at diagnosis, autoimmune disease diagnosis, tumor sequence, and history of radiotherapy or surgery. Given that SEER cancer registries and Medicare claims are completed based on data collected in clinical settings, demographic information, such as race and sex, reflect the information provided by the patient.^17^ We used the 2015 SEER-Medicare Census Zip Code file to derive quartiles of zip code–level median per capita income and percentage of residents who were high school graduates. The modification by Warren et al^14^ of the Charlson Comorbidity Index (CCI) was used to quantify severity of preexisting comorbidities. The primary outcome was overall survival, defined as time from the index date until death from any cause, with patients censored at the end of the study (December 31, 2017).

### Statistical Analysis

Statistical analysis was performed from September 9, 2019, to February 20, 2021. The Kaplan-Meier method was used to compare overall survival between female and male patients. Differences in time-to-event outcomes between sexes were evaluated using the log-rank test. Effect modification by sex was tested using a Cox proportional hazards regression model with an interaction term for each subgroup and sex. Sex differences in mortality were examined using Cox proportional hazards regression models, which adjusted for age at index date, CCI, cancer stage at the time of diagnosis, and autoimmune disease diagnosis. All statistical analyses were 2-sided and performed at the 5% significance level using SAS, version 9.4 statistical software (SAS Institute Inc). To evaluate the effectiveness of each treatment in comparison with another among female and male patients, we set “anti–PD-1 with prior ipilimumab use” as the reference group to calculate the hazard ratio (HR) for the other 2 treatment subgroups within each sex.

## Results

### Patients

In total, 1369 patients (982 men [71.7%] and 387 women [28.3%]; median age, 75 years [IQR, 69-82 years]; 165 [12.1%] with ≥1 autoimmune diagnosis) with a diagnosis of melanoma met study inclusion criteria (Table 1). A total of 1204 patients (87.9%) were receiving anti–PD-1 therapy, while the remaining 165 patients (12.1%) were receiving combination therapy.

**Table 1.** Demographic and Clinical Characteristics of Patients With Advanced Melanoma

Characteristic	Patients, No. (%)	
	Female (n = 387)	Male (n = 982)
Immune checkpoint inhibitors		
Anti–PD-1 therapy	347 (89.7)	857 (87.3)
Nivolumab plus ipilimumab combination therapy	40 (10.3)	125 (12.7)
History of ipilimumab		
Yes	153 (39.5)	374 (38.1)
No	234 (60.5)	608 (61.9)
Age at index time (administration of the immunotherapy), y		
<70	109 (28.2)	276 (28.1)
70-79	151 (39.0)	407 (41.4)
≥80	127 (32.8)	299 (30.4)
Year of diagnosis		
1991-2000	28 (7.2)	80 (8.1)
2001-2005	47 (12.1)	132 (13.4)
2006-2010	96 (24.8)	236 (24.0)
2011-2015	216 (55.8)	534 (54.4)
Autoimmune disease diagnosis		
0	320 (82.7)	884 (90.0)
≥1	67 (17.3)	98 (10.0)
Cancer history		
Melanoma only	193 (49.9)	449 (45.7)
History of other primary cancer	194 (50.1)	533 (54.3)
Charlson Comorbidity Index		
0	276 (71.3)	685 (69.8)
≥1	111 (28.7)	297 (30.2)
Cancer stage at diagnosis		
0-II or unknown	284 (73.4)	700 (71.3)
III	67 (17.3)	196 (20.0)
IV	36 (9.3)	86 (8.8)
History of radiotherapy		
No radiotherapy or surgery	338 (87.3)	857 (87.3)
Radiotherapy and surgery (once each)	49 (12.7)	125 (12.7)
SEER region		
Northeast	82 (21.2)	190 (19.3)
South	81 (20.9)	203 (20.7)
North central	26 (6.7)	80 (8.1)
West	198 (51.2)	509 (51.8)
Zip code–level, median (IQR)		
Per capita income, $	31 130 (23 773-42 575)	31 180.5 (24 464-41 622)
High school graduate, %	23.9 (16.6-31.2)	23.8 (16.4-31.3)

Abbreviations: PD-1, programmed cell death protein 1; SEER, Surveillance, Epidemiology, and End Results.

Female and male patients were similar across all clinical and demographic characteristics except in the prevalence of autoimmune disease. A significantly higher proportion of women than men (67 of 387 [17.3%] vs 98 of 982 [10.0%]) had a diagnosis of autoimmune disease (Table 1).

### Mortality by Sex

The distribution of death was comparable between women and men among those patients receiving anti–PD-1 therapy, regardless of their prior use of ipilimumab (prior use of ipilimumab: women, 45 of 113 [39.8%] and men, 102 of 249 [41.0%]; and no prior use of ipilimumab: women, 95 of 234 [40.6%] and men, 274 of 608 [45.1%]) (eTable in the Supplement). Conversely, there was imbalance in the distribution of death between female and male patients receiving the nivolumab plus ipilimumab combination therapy (26 of 40 [65.0%] vs 50 of 125 [40.0%]).

**Figure 2.**
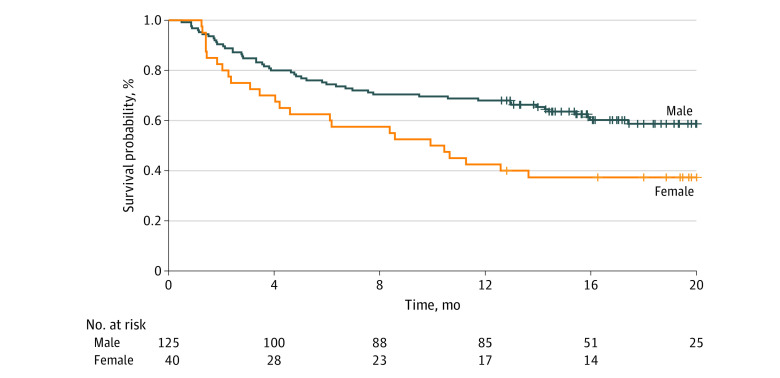
Overall Survival for Patients Receiving Nivolumab Plus Ipilimumab Combination Therapy, by Sex Median survival was reached for female patients receiving combination therapy (10.2 months [95% CI, 4.6-23.9 months]), while more than 50% of male patients receiving combination therapy survived by the end of the study period (75 of 125 [60.0%]).

Patients were followed up for up to 24 months. Median survival was not reached for either sex in the entire patient cohort, and there was not a significant difference in survival between men and women (eFigure 1 in the Supplement). When survival analyses were performed for the 3 predefined subgroups, median survival was reached only for female patients receiving combination therapy (10.2 months; 95% CI, 4.6-23.9 months) (Figure 2). More than 50% of patients receiving anti–PD-1 therapy survived by the end of the study period, regardless of their prior ipilimumab use (no ipilimumab history, 473 of 842 [56.2%]; and ipilimumab history, 215 of 362 [59.4%]). No significant difference in survival was seen between male and female patients receiving anti–PD-1 therapy (eFigures 2 and 3 in the Supplement).

There was evidence of effect modification by sex among patients treated with ICIs. The interaction term between sex and the subgroup classification, which accounted for both the last ICI the patient received and their prior ipilimumab use, was significant (Wald χ^2^ = 9.48; *P* = .009). Table 2 shows that, for women with prior ipilimumab use, combination therapy was associated with 2.82 times higher hazard of mortality (95% CI, 1.73-4.60) than anti–PD-1 therapy. On the other hand, no statistically significant difference was seen in mortality hazards between anti–PD-1 therapy and combination therapy for men, regardless of prior ipilimumab use. Furthermore, Table 3 shows that women receiving combination therapy had 2.06 times higher hazard (95% CI, 1.28-3.32; *P* = .003) of overall mortality compared with their male counterparts, after adjusting for age at index date, CCI, cancer stage, and autoimmune disease diagnosis. No statistically significant difference was observed between women and men receiving anti–PD-1 therapy with (HR, 0.97 [95% CI, 0.68-1.38]; *P* = .85) or without prior ipilimumab use (HR, 0.85 [95% CI, 0.67-1.07]; *P* = .16).

**Table 2.** Multivariable Cox Proportional Hazard Regression Analysis of Overall Mortality Hazard Ratio

Subgroup	Hazard ratio (95% CI)	
	Female patients	Male patients
Anti–PD-1		
With prior ipilimumab use	1 [Reference for other female subgroups]	1 [Reference for other male subgroups]
Without prior ipilimumab use	1.06 (0.74-1.51)	1.21 (0.96-1.53)
Combination therapy with prior ipilimumab use	2.82 (1.73-4.60)	1.32 (0.94-1.87)

Abbreviation: PD-1, programmed cell death protein 1.

**Table 3.** Multivariable Cox Proportional Hazard Regression Analysis of Overall Mortality Hazard Ratio

Subgroup	Female vs male patients, HR (95% CI)	*P* value
Anti–PD-1 therapy		
With ipilimumab history	0.97 (0.68-1.38)	.85
Without ipilimumab history	0.85 (0.67-1.07)	.16
Combination therapy with ipilimumab history	2.06 (1.28-3.32)	.003

Abbreviations: HR, hazard ratio; PD-1, programmed cell death protein 1.

## Discussion

This population-based cohort study showed that female sex was associated with a 2-fold increased hazard of mortality compared with male sex among those receiving nivolumab plus ipilimumab combination therapy. In addition, combination therapy was associated with 2.82 times higher hazard of mortality than anti–PD-1 therapy for women with prior ipilimumab use. These novel findings suggest that, for women with a prior history of ipilimumab, treatment with anti–PD-1 therapy may be preferable to combination therapy, whereas for men, it is unclear which treatment is better.

To our knowledge, analyses of the association of sex with the effectiveness of ICIs in advanced melanoma have been conducted only using clinical trial data. Clinical trials are at risk for having nongeneralizable results owing to referral bias and strict inclusion criteria. By using the SEER-Medicare linked data, we included older patients and those with autoimmune diseases and minimized those biases. By design, this population-based cohort study ensured that the patient cohort was representative of patients with advanced melanoma in real life.

### Potential Mechanism of Action

The sex dimorphism seen in ICI response among the patients receiving combination therapy may be explained by the sex-associated molecular difference in tumors, particularly in tumor mutation burden (TMB) and neoantigens. Immune checkpoint inhibitors are designed to elicit T-cell responses directed at immunogenic neoantigens. These neoantigens exist owing to the mutations in the coding region of the tumor genome.^18^ Therefore, if the TMB is high and, in turn, if antigens are produced in a large quantity, the effectiveness of the immunotherapy could be optimized.^19^ Studies evaluating The Cancer Genome Atlas cancers identified significant sex-mutation differential in melanoma, with a lower TMB in female patients.^20,21^ Furthermore, Ye et al^21^ demonstrated a survival disadvantage in female patients with melanoma receiving ICIs, drawing an association between TMB and ICI effectiveness. A recent report showed that the tumor-expressed antigens in women are also less likely to be presented to the immune system by the major histocompatibility complex.^22^ Stronger immune selection during tumorigenesis in women is posited as the reason why immunogenecity of the remaining tumor cells tends to be low.^23^ Both the low level of TMB and immunologically invisible neoantigens may explain the reduced association of ICI treatment with outcomes among women receiving combination therapy in our study.

In addition to the tumor mutations, the change in the estrogen signaling pathway with aging may also be associated with the varying levels of ICI treatment response observed between women and men with melanoma who are receiving combination therapy. The expression of estrogen receptor (ER)–β is directly related to the endogenous estrogen level.^24^ Studies demonstrated that when ER-β expression decreases owing to the reduced production of estrogen, cellular proliferation becomes uncontrolled, leading to metastasis.^24,25^ The decrease in ER-β expression and the consequent disturbance in the balance between ER-α and ER-β are thought to be associated with resistance to the ER antagonist tamoxifen in breast cancer cells.^6,26,27^ Given that the patient population comprised Medicare beneficiaries, all women were in their postmenopausal period and were likely to have experienced a decrease in ER-β expression. Although evaluation of the ER-β expression level between postmenopausal women and older men is warranted, we posit that unfavorable mortality among women receiving combination therapy may be due to this change in the estrogen signaling pathway.

It is unknown why sex-specific survival was seen in the combination therapy group but not in the anti–PD-1 therapy group. Although the association of sex with immunotherapy effectiveness has been examined in multiple meta-analyses, none has tested this hypothesis specifically for patients with advanced melanoma receiving anti–PD-1 therapy or combination therapy, to our knowledge. The meta-analyses either combined multiple types of patients with cancer to investigate the sex differential in survival by treatment group,^28,29^ or vice versa.^21,29^ Therefore, it is impossible to assess the external validity of our study results. We speculate that the difference in the mechanism between PD-1 inhibitors and CTLA-4 inhibitors may have resulted in the difference in the association of sex with outcomes that we see between the 2 immunotherapy groups. Programmed cell death protein 1 inhibitors and CTLA-4 inhibitors work at different points in T-cell regulation, and they may interact differently with sex-specific biomarkers. Future studies are warranted to test this hypothesis.

### Clinical Implications

The effect modification by sex among patients treated with ICIs is not a phenomenon specific to patients with melanoma receiving combination therapy. The meta-analysis by Conforti et al^28^ of 20 phase 2 and phase 3 trials among patients with multiple advanced cancers, mostly including melanoma and non–small-cell lung cancer (NSCLC), demonstrated a statistically significant difference in effectiveness between female and male patients treated with a CTLA-4 inhibitor and a PD-1 inhibitor. For female patients receiving ICI treatment, the pooled mortality HR was 0.86 (95% CI, 0.79-0.93), while for male patients, the pooled mortality HR was 0.72 (95% CI, 0.65-0.79), and the difference was significant (*P* = .002). Female patients with NSCLC had a significant overall survival advantage associated with PD-1 inhibitor or programmed death-ligand 1 inhibitor treatment combined with chemotherapy compared with treatment with chemotherapy alone, suggesting that an ICI combined with chemotherapy may bring out the optimal outcome for female patients with NSCLC.^28^ A recent, real-world data analysis by Hadash-Bengad et al^30^ shares the same suggestion for metastatic melanoma treatment. In their analysis, the authors observed a significant improvement in progression-free survival and clinically meaningful improvement in overall survival among patients who were treated with chemotherapy after ICI-based immunotherapy. These findings illustrate that treating metastatic melanoma with chemotherapy combined with ICIs may enhance the immune response and optimize the immunotherapy treatment, particularly for women.

Despite the accumulating evidence of the potential role played by sex in drug effectiveness owing to the biological differences between men and women, the effectiveness of new therapeutic approaches is rarely examined by sex.^31,32^ This lack of attention on the association of sex with the effectiveness of ICI-based immunotherapy may have significant negative consequences, especially because these treatments are associated with high toxicity and high treatment cost. For future trials, it would be crucial to examine effect modification by sex.

### Limitations

This analysis has several limitations. First, in the SEER-Medicare data set, we did not have access to clinical data that would have provided patients’ cancer stage at the time of ICI administration, cause of mortality, and regimen dose. We assumed that the patients in our analytic cohort had progressed to stage III or IV melanoma by the index date, given that these therapies were approved for those with unresectable (stage III) or metastatic (stage IV) melanoma, and were receiving the same dose. Second, our study included 165 patients receiving combination therapy, with only 40 of them being women (Table 1). This relatively small sample size may have introduced selection bias. Nonetheless, we observed that the mortality pattern in treatment groups followed the pattern seen in clinical trials, with patients receiving combination therapy having higher mortality than those receiving anti–PD-1 therapy (eFigure 3 in the Supplement). Third, an observational study is subject to residual confounding owing to the lack of randomization. Given that combination therapy is associated with higher toxicity than monotherapy, physicians are more likely to prescribe combination therapy to younger and/or healthier patients. Although controlling for patient’s age, CCI, cancer stage, and autoimmune disease diagnosis may have helped reduce selection bias, confounders that were not observed in our analyses may have been associated with immunotherapy outcomes. Fourth, the potential residual effects of prior treatments may have been a factor associated with overall survival. There were 26 of 165 patients in the combination therapy group (15.8%) who were receiving anti–PD-1 therapy prior to switching to combination therapy. Recent reports demonstrate that approximately one-third of the patients who failed to respond to anti–PD-1 monotherapy responded to combination therapy. Patients who were refractory to ipilimumab were also observed to respond to combination therapy in clinical settings.^33,34,35^ Therefore, we treated the patients receiving combination therapy who switched from an anti–PD-1 therapy the same as those who switched from ipilimumab. However, there are still many uncertainties around the mechanism of action, and this group should be investigated separately in a study with a larger sample size.

Although literature provides strong evidence that immunotherapy may not be as effective for female patients with melanoma as it is for their male counterparts owing to biological differences, we cannot completely rule out the possibility that the differences in outcomes may be due to differences in behavioral patterns (eg, smoking, outdoor activities, and health care resource use). It would be imperative to replicate this study with a larger patient population, including younger cohorts to examine whether genetic and hormonal factors play a role in ICI response. Furthermore, it is not possible for us to understand why the sex differential was observed only among patients receiving combination therapy and not among those receiving anti–PD-1 therapy. Future research using clinical and molecular-level data is warranted to further understand the mortality pattern observed in this study.

## Conclusions

In this population-based cohort study, we observed that female patients with advanced melanoma treated with nivolumab plus ipilimumab combination therapy had an overall mortality risk 2 times higher than their male counterparts after adjusting for known confounding variables. Further research is warranted to validate our findings, to explore potential biological mechanisms, and to design optimal treatment strategies tailored to each patient.

## Notes

Supplement. **eTable.** Distribution of Death Between Female and Male Advanced Melanoma Patients, by Subgroup **eFigure 1.** Overall Survival for the Entire Patient Cohort, by Sex **eFigure 2.** Overall Survival for Anti–PD-1 Therapy Patients Without Prior Use of Ipilimumab, by Sex **eFigure 3.** Overall Survival for Anti–PD-1 Therapy Patients With Prior Use of Ipilimumab, by Sex
