# Supplementary material for: Association Between Sex and Immune Checkpoint Inhibitor Outcomes for Patients With Melanoma
Source: JAMA Netw Open. 2021 Dec 2;4(12):e2136823. doi: 10.1001/jamanetworkopen.2021.36823 (PMC8640892; doi:10.1001/jamanetworkopen.2021.36823)
Supplement: Supplement. — eTable. Distribution of Death Between Female and Male Advanced Melanoma Patients, by Subgroup eFigure 1. Overall Survival for the Entire Patient Cohort, by Sex eFigure 2. Overall Survival for Anti–PD-1 Therapy Patients Without Prior Use of Ipilimumab, by Sex eFigure 3. Overall Survival for Anti–PD-1 Therapy Patients With Prior Use of Ipilimumab, by Sex [file jamanetwopen-e2136823-s001.pdf]

Supplementary Online Content

Jang SR, Nikita N, Banks J, et al. Association between sex and immune checkpoint inhibitor outcomes for patients with melanoma. *JAMA Netw Open.* 2021;4(12):e2136823. doi:10.1001/jamanetworkopen.2021.36823

eTable. Distribution of Death Between Female and Male Advanced Melanoma Patients, by Subgroup
eFigure 1. Overall Survival for the Entire Patient Cohort, by Sex
eFigure 2. Overall Survival for Anti–PD-1 Therapy Patients Without Prior Use of Ipilimumab, by Sex
eFigure 3. Overall Survival for Anti–PD-1 Therapy Patients With Prior Use of Ipilimumab, by Sex

This supplementary material has been provided by the authors to give readers additional information about their work.

eTable. Distribution of Death Between Female and Male Advanced Melanoma Patients, by Subgroup

		Death	
**Subgroup**	**Sex**	**Yes, n (%)**	**No, n (%)**
Anti-PD1 with Ipilimumab History	Female	45 (39.8%)	68 (60.2%)
	Male	102 (41.0%)	147 (59.0%)
Anti-PD1 without Ipilimumab History	Female	95 (40.6%)	139 (59.4%)
	Male	274 (45.1%)	334 (54.9%)
Combination Therapy with Ipilimumab History	Female	26 (65.0%)	14 (35.0%)
	Male	50 (40.0%)	75 (60.0%)

© 2021 Jang SR et al. *JAMA Network Open.*

eFigure 1. Overall Survival for the Entire Patient Cohort, by Sex

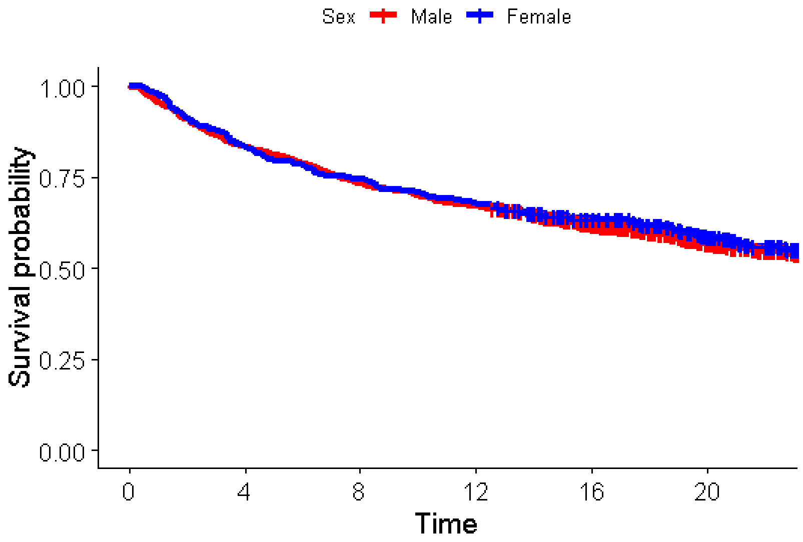

No. at Risk

Male	982	820	719	659	512	365
Female	387	323	288	263	222	160

The unit of measure for the x-axis (ie, time) is months, and the unit of measure for the y-axis (ie, survival probability) is percent.

© 2021 Jang SR et al. *JAMA Network Open*.

eFigure 2. Overall Survival for Anti–PD-1 Therapy Patients Without Prior Use of Ipilimumab, by Sex

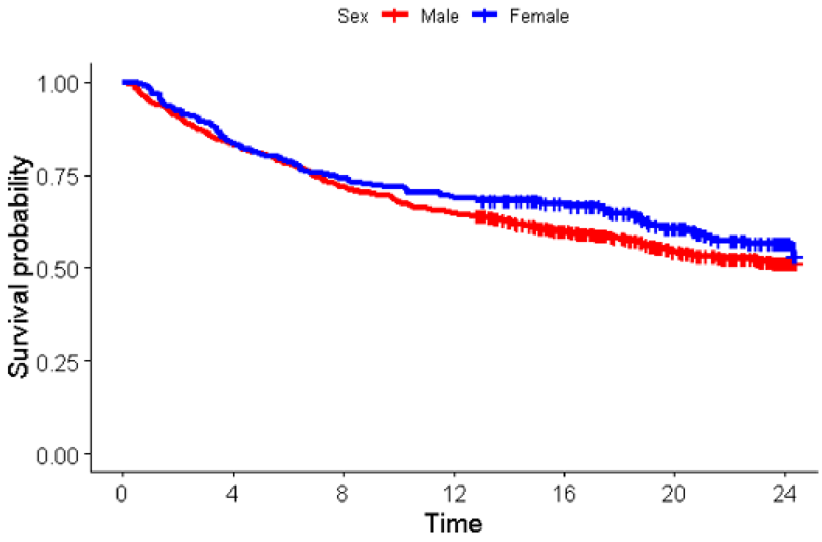

No. at Risk

Male	608	507	436	394	303	210	93
Female	234	195	173	162	138	97	38

The unit of measure for the x-axis (ie, time) is months, and the unit of measure for the y-axis (ie, survival probability) is percent.

© 2021 Jang SR et al. *JAMA Network Open*.

eFigure 3. Overall Survival for Anti–PD-1 Therapy Patients With Prior Use of Ipilimumab, by Sex

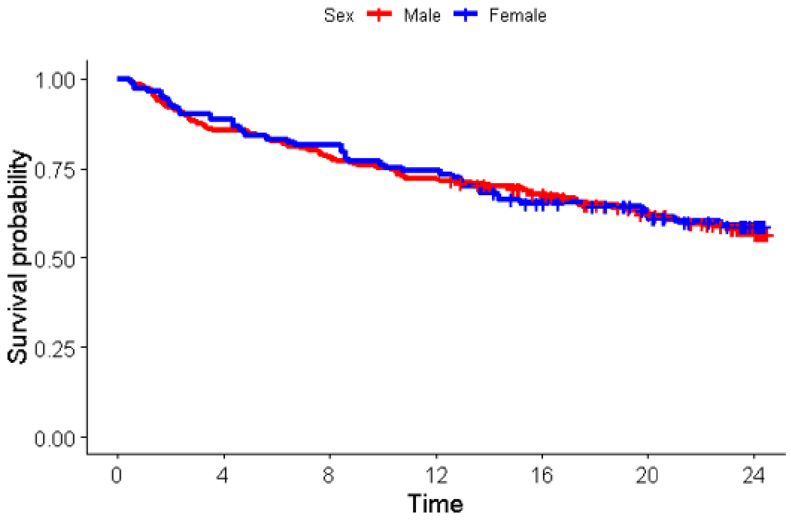

No. at Risk

Male	244	213	195	180	158	130	77
Female	113	100	92	84	70	58	35

The unit of measure for the x-axis (ie, time) is months, and the unit of measure for the y-axis (ie, survival probability) is percent.

© 2021 Jang SR et al. *JAMA Network Open*.
